# Adipose Dysfunction Caused by Obesity and Radiation Therapy Rewires the Prostate Stroma Toward Tumor Progression

**DOI:** 10.3390/cells15151387

**Published:** 2026-07-31

**Authors:** Simran Takkar, Louise Monga-Wells, Arpita Chatterjee, Subodh M. Lele, Rebecca E. Oberley-Deegan

**Affiliations:** 1 Department of Biochemistry and Molecular Biology, University of Nebraska Medical Center, Omaha, NE 68198, USA; stakkar@unmc.edu (S.T.); louise.monga@unmc.edu (L.M.-W.); arpita.chatterjee@unmc.edu (A.C.); 2 Department of Pathology, Microbiology, and Immunology, University of Nebraska Medical Center, Omaha, NE 68198, USA

**Keywords:** obesity, adipose tissue, radiation, tumor microenvironment, fibroblast activation, cytokines, inflammation, prostate cancer

## Abstract

Obesity is associated with chronic adipose dysfunction characterized by oxidative stress, inflammation, senescence, and fibrosis, which can promote tumor progression. In prostate cancer, periprostatic adipose tissue may directly influence the prostate microenvironment. Radiation therapy is widely used in prostate cancer, but radiation-induced adipose dysfunction in obesity and its impact on the prostate microenvironment remain poorly understood. In the present study, we investigated the impact of obese and irradiated obese adipose microenvironments on prostate stromal remodeling, as well as the activation of prostate fibroblasts mediating prostate cancer progression. We utilized a high-fat diet obesity model with localized adipose irradiation in animal and in vitro studies using obese and irradiated obese adipocytes. Prostates from obese and irradiated obese mice exhibited epithelial hyperplasia, increased stromal activation markers, oxidative damage, and senescence. Interestingly, radiation maintained the obesity-induced pathological behavior in the prostate, rather than elevating it. The conditioned media from obese and irradiated obese adipocytes induced stromal activation markers, senescence, extracellular H_2_O_2_ production, pro-survival signaling, and inflammation. Notably, the only significant changes observed with the addition of radiation to obesity were enhancement of fibrosis-associated features and infiltration of CD4^+^ T cells. Functionally, prostate myofibroblasts or senescent fibroblasts promoted prostate cancer migration and induced epithelial-to-mesenchymal transition and elevated pro-tumorigenic pathways. Cytokine profiling identified elevated levels of CXCL10 and CXCL11 from myofibroblasts, and pharmacological inhibition of CXCR3 significantly reduced prostate cancer migration, implicating this signaling axis in activated fibroblast-driven tumor-promoting crosstalk. Collectively, these findings demonstrate obesity-associated adipose dysfunction reprograms the prostate microenvironment toward a pro-tumorigenic state, while radiation sustains rather than markedly amplifies these pathological changes, identifying obese adipose-stromal crosstalk and the CXCL10/CXCL11-CXCR3 axis as potential therapeutic targets to inhibit prostate cancer progression.

## 1. Introduction

Obesity is a major health concern globally and is a risk factor for various diseases, including cardiovascular disease, cancer, metabolic syndromes, poor bone health, and diabetes [1,2,3]. During obesity, adipose tissue undergoes chronic remodeling characterized by oxidative stress, inflammation, extracellular matrix deposition, and immune infiltration [4,5,6]. Worldwide, approximately 3.6% of cancers (481,000 new cases annually) are linked to elevated body mass [7]. Indeed, studies have shown the role of obesity in promoting cancers, including breast, renal, prostate, and colorectal cancers [8,9,10,11,12,13]. Mechanistically, obesity promotes carcinogenesis by altering the tumor microenvironment (TME) through systemic inflammation, endocrine disturbances, immune dysregulation, metabolic remodeling, and oxidative stress, all of which promote cancer [2,14]. Subsequently, dysfunctional, oxidatively stressed obese adipose tissue secretes chemokines or cytokines that can influence neighboring organs [15,16]. Anatomically, prostates are surrounded by periprostatic adipose tissue, and we speculate that the altered obese adipose tissue can influence the microenvironment of the prostate promoting epithelial hyperplasia or stromal remodeling that supports prostate cancer progression [17].

Radiation therapy is a standard treatment for various cancers such as prostate, head and neck, anal, breast, and rectal cancer [18,19,20,21,22,23,24]. It is an effective treatment since it delivers the maximum dose to the tumor, and its impact is more deleterious to fast-dividing cells such as cancer [25,26]. Unfortunately, radiotherapy in pelvic cancers, such as prostate cancer, can have a detrimental impact on the surrounding normal tissues or organs. Recent studies from our lab show that radiation enhances the tissue dysfunction of the surrounding adipose tissue present in the pelvic region of normal or obese mice [4,27]. Thus, these findings suggest that obese dysfunctional adipose tissue alone or in the presence of radiation can sustain a pathological microenvironment that could promote disease progression. Patients with obesity who have pelvic cancer, such as prostate cancer, undergoing radiation treatment suffer increased likelihood of tumor recurrence and higher metastatic potential [28,29]. Despite these observations, the combined effect of obesity and radiation-induced adipose dysfunction on the prostate microenvironment remains poorly understood.

The TME plays a major role in the progression and development of cancer and therapeutic resistance [30,31,32]. Stromal fibroblasts are abundant and are key regulators of tissue remodeling and cancer progression [30,32]. Activated fibroblasts acquire a myofibroblast phenotype that is characterized by elevated expression of stromal markers [33]. In addition, persistent oxidative stress or growth factors drive fibroblast activation, which in turn enhances cellular senescence, inflammation, and fibrotic remodeling leading to a tumor-promoting niche [33]. These activated fibroblasts also promote tumor progression through the secretion of chemokines and extracellular matrix proteins that support the survival, proliferation, and migration of cancer cells [34,35,36]. For instance, activated fibroblasts, or myofibroblasts, promote different cancers including prostate, hepatocellular, glioblastoma, pancreatic, and colon cancer by secreting chemokines, such as CXCL11, CCL5, CCL2, CXCL10, and CCL12 [37,38,39,40,41]. However, the role of dysfunctional obese adipose in the context of radiation in promoting a pro-tumor microenvironment, particularly in driving prostate remodeling or fibroblast activation, is not known. Given the close anatomical proximity between adipose tissue and the prostate gland, it is important to determine the consequences of obesity-associated adipose dysfunction along with radiation on prostate tissue remodeling.

In this study, we investigated the influence of obesity and radiation-induced adipose dysfunction on the prostate microenvironment and the mouse prostate fibroblast. Using in vitro and in vivo models, we have demonstrated that obesity-associated adipose dysfunction promotes stromal remodeling, oxidative stress, fibroblast activation, and senescence leading to a pro-inflammatory and pro-fibrotic stromal niche within the prostate microenvironment. Overall, radiation exposure of obese adipose tissue sustains these pathological changes without further exacerbating them. Furthermore, prostate fibroblasts that transitioned into myofibroblasts under obesity-associated or radiation-induced obese adipose conditions enhanced prostate cancer migration by secreting pro-inflammatory chemokines, indicating that normal prostate fibroblasts, once transitioned into myofibroblasts, are key drivers of prostate cancer progression.

## 2. Material and Methods

### 2.1. Primary Prostate Fibroblast Isolation and Cell Culture

C57BL/6 mice (4–5 weeks old from Jackson Laboratories, Bar Harbor, ME, USA) were used for the isolation of mouse primary fibroblasts (MPFs), as previously described [33,42,43]. Isolated prostates were minced thoroughly with a scalpel and digested in 5 mg/mL collagenase type1 (Cat. 17100017, Thermo Fisher, Waltham, MA, USA) at 37 °C for 30 min. Tissue fragments and liberated cells were then cultured in the Dulbecco Minimal Essential media (DMEM) containing 1% non-essential amino acids (NEAA), 1% penicillin/streptomycin (P/S), and 10% fetal bovine serum (FBS) for 2–3 weeks at 37 °C. After culturing the cells for 5–6 days, only fibroblasts remained. Normal immortalized human prostate fibroblast cells (HPFs), P3158 cells, were maintained in RPMI-1640 (Cat#SH30027.01) media containing 10% FBS, and 1% P/S. P3158 were a kind gift from Dr. J Tyson McDonald and were immortalized by the pBABE-hygro-hTERT plasmid cat #1773 (Addgene, Watertwon, MA, USA).

### 2.2. Cell Culture Radiation and Collection of the Conditioned Media

3T3-L1, mouse embryonic fibroblast-derived preadipocyte cell line was purchased from ATCC (CL-173, Manassas, VA, USA). To mimic obese conditions, we exposed differentiated 3T3-L1 to the fatty acid cocktail containing 0.75 mM oleic acid (Cat# O3008, Millipore Sigma, St. Louis, MO, USA), 0.75 mM linolenic acid (Cat# L9530, Millipore Sigma), and 0.5 mM palmitic acid (Cat# P5585, Millipore Sigma), as described previously from our laboratory study and by others [4,44]. The obese 3T3-L1 adipocytes were sham (0 Gy) or radiated with 3 Gy for 3 consecutive days (fractionated exposure, 1.2 Gy/min, 160 KV) using an irradiator box (RadSource 2000 X-ray, Buford, GA, USA). The CM was collected from obese or irradiated obese 3T3-L1 adipocytes 48 h post-radiation. The collected CM was centrifuged at 500× *g* for 5 min to remove any debris and stored at −80 °C. The fatty acid cocktail was replaced with 0.2% FBS containing DMEM media in obese 3T3-L1 adipocytes before radiation. The RadSource 2000 X-ray Box Irradiator is calibrated annually using an ion chamber.

### 2.3. Diet Induced Obesity Model and In Vivo Radiation Treatments

4–5-week-old C57BL/6 male mice were randomly divided into two groups and fed with either normal diet (ND 7012 Teklad, Inotiv, Madison, WI, USA) or 60% high-fat diet (HFD) [D12492i Research Diets, 60% kcal lard fat, with macronutrient composition of protein (Lactic Cystine L, Casein), fiber (Solka Floc FCC200), carbohydrate (sucrose fine granulated, Lodex 10), and vitamin (Choline Bitartrate)] as described in our previous study [4]. The ND and HFD fed mice were housed in accordance with the Guide for the Care and Use of Laboratory Animals by the National Institute of Health at the University of Nebraska Medical Center (UNMC). The mice were exposed to a 12 h light/dark cycle and fed ad libitum. All experiments were approved by UNMC Institutional Animal Care and Use Committee (IACUC; original 23-039-00-FC was renewed to 25-100-02-FC on 2 February 2026). This study was conducted in compliance with the ARRIVE guidelines. After 16 weeks of ND or HFD, the mice were randomly divided into three groups using a random number generator: ND *n* = 5, HFD *n* = 6, and HFD mice that received radiation directly at both the fat pads *n* = 8. For radiation treatments, the mice were anesthetized by isoflurane and irradiated using a small animal radiation research platform (SARRP, Xstrahl Life Sciences, Suwanee, GA, USA) irradiator and HFD mice were radiated directly at both fat pads using a 5 mm × 5 mm collimator based on CT imaging with 7.5 Gy of radiation for 5 consecutive days (total dose of 37.5 Gy, 3 Gy/min X-rays) only a small portion of each fat pad was irradiated. Mice were monitored weekly until the end of the experiment. Two months post-radiation, the adipose and prostate tissues were collected from all three groups. Investigators were blinded during IHC image capturing, analysis, and pathological assessments.

### 2.4. Isolation of Mouse Primary Adipocytes

Primary adipocytes were isolated from fat pads as reported previously [33]. C57BL/6 mice exposed to either ND or HFD for 16 weeks or HFD mice that received radiation directly at the fat pads were used to isolate the gonadal fat pads. The fat tissue from the respective groups was agitated until the solution turned cloudy at 37 °C for 45 min in collagenase I. The cells were then centrifuged at 400× *g* for 5 min. The lipid layer after centrifugation was discarded and pelleted cells were cultured in DMEM supplemented with 10% FBS, 1% P/S, and 1% NEAA in a 6-well plate in a humidified incubator at 37 °C. The next day the floating fat cells were moved to a new well by agitation. The following day, the fat floating on top was removed from each group and at this time the mature cells (primary adipocytes) had settled to the bottom of the well. Fresh media (DMEM containing 0.2% FBS, 1%P/S, and 1%NEAA) were added to these mature cells, and the CM was collected 48 h later from these cells, as described in Figure 4A. The collected CM was centrifuged at 400× *g* for 5 min to remove particulate debris and stored at −80 °C.

### 2.5. Cell Size Estimation

The isolated normal mouse prostate fibroblasts were seeded at a concentration of 50,000 cells per well. The following day, fibroblasts were exposed to conditioned media (CM) collected from the cultured adipose isolated from ND, HFD, and HFD mice that received radiation directly at the fat pads for 4 days, with media replenishment on day 2 (48 h). At the end of the experiment, 3–5 images were captured per group using the Leica inverted phase-contrast microscope. The cell size was measured from the captured images by marking the cell borders using ImageJ software (ImageJ 1.54f, National Institute of Health, USA). The larger cell area correlates with prostate fibroblast activation. GraphPad Prism v10 software was used to analyze the cell size in the respective groups.

### 2.6. Senescence Assay

HPFs or MPFs (50,000/per well) were seeded in a 6-well plate. The next day, the respective seeded cells were exposed to either the CM collected from ex vivo adipose tissue from ND, HFD, and HFD mice that received radiation directly at the fat pads or the CM collected from obese or irradiated obese 3T3-L1 adipocytes (3 Gy) for 4 days, with media replacement on day 2. On day 4, 4% paraformaldehyde in PBS was used to fix cells for 5–6 min. After fixation, cells were washed with PBS 2–3 times before incubation with senescence-associated beta galactosidase (SA-β-Gal) staining solution as previously reported [4,33]. After 72 h of incubation, images of cells were taken using a brightfield Olympus IX81 inverted microscope (Olympus America Inc., Melville, NY, USA) at 200×. A total of 3–5 images were taken per group. Cells exhibiting a green-blue staining (positive β-Gal cells) were counted per image and the data are represented as the positive number of cells per image in each group.

### 2.7. Cell Viability

HPFs were seeded in a 6-well plate at a concentration of 50,000 cells/per well for 24 h. The next day, HPFs were incubated with CM from obese or irradiated obese 3T3-L1 adipocytes for 4 days, with media replenishment on day 2. On day 4, the media and trypsinized cells were combined and centrifuged at 400× *g* for 5 min. The pellet was resuspended in 200 µL of fresh RPMI media, and a small aliquot was mixed at a 1:1 ratio with trypan blue. The dead and living cells were counted using a hemocytometer.

### 2.8. Amplex Red Assay

50,000 HPFs were seeded per well in a 6-well plate. The next day, the cells were exposed to CM from obese or irradiated obese 3T3-L1 adipocyte CM for 4 days, with a media replacement at 48 h. On day 4, once the HPFs became activated, the CM was removed and cells were washed thoroughly 3 times with PBS. Twenty-four and 48 h after adding the fresh media, the CM was collected from the HPFs. The Amplex Red H_2_O_2_ assay (Cat #A36006, Invitrogen, Carlsbad, CA, USA) was used to estimate the extracellular H_2_O_2_ in the freshly collected CM. Absorbance was read from 570 to 590 nm.

### 2.9. Immunofluorescence

PC3 cells were incubated in standard RPMI media, CM of normal MPF, MPF incubated in obese or irradiated obese 3T3-L1 adipocyte CM for 48 h. After 48 h, PC3 cells were trypsinized and cytospun onto glass slides. The cells were fixed for 10 min in 4% paraformaldehyde and incubated in 0.5% triton-X in PBS for 10 min. Following fixation, the permeabilized cells were washed in PBS three times for 5 min and blocked in 10% goat serum for 1 h at room temperature. Next, cells were incubated with primary antibodies against vimentin (1:100, Cat. #5741S), Cell Signaling Technology (CST, Danvers, MA, USA) or E-cadherin (1:100, Cat#3195S, CST) at 4 °C. The following day, cells were washed in PBS three times for 5 min and incubated for 1 h in secondary antibody anti-rabbit 488 (1:1000, Cat#A-11008, Invitrogen) at room temperature. At least 5–6 images were captured for each replicate and averaged for quantification. ImageJ software was used to measure the mean fluorescence intensity.

### 2.10. Immunohistochemistry and Histology

Isolated tissues were sectioned and then H&E or immunohistochemical staining was performed as described previously [4,45]. Briefly, slides with paraffin-embedded tissue were deparaffinized by xylene twice and then rehydrated in the graded alcohols, followed by antigen retrieval at 95 °C in sodium citrate buffer (0.01 M sodium citrate, pH 6, for 20 min). The slides were then washed, blocked, and incubated at 4 °C overnight with primary antibodies. The next day slides were washed with PBS 3 times for 10 min. Washed slides were incubated at room temperature for 2 h with secondary antibodies in the dark. Fluorescent secondary antibodies used in the present study are anti-rabbit 488 (1:1000, Cat#A-11008, Invitrogen), anti-donkey goat AlexaFluor 555 (1:1000, Cat#A21432, Invitrogen), and anti-mouse AlexaFluor 488 (1:1000, Cat#A11001, Invitrogen).

Primary antibodies used: To measure oxidative stress mouse anti-4-hydroxynonenal (1:400, Cat#BS-6313R, Thermo Fisher Scientific, Waltham, MA, USA) and mouse anti-DNA/RNA damage (1:400, Cat#AB15A3, Abcam, Cambridge, UK) were used. A Mouse on Mouse kit (Vector Laboratories, Newark, CA, USA, FMK-2201) was utilized to minimize non-specific staining. Stromal remodeling activation markers were quantified by staining the prostate tissue sections with α-SMA (1:200, Cat#NB300-978 Invitrogen), fibroblast activation protein (FAP) (1:200, Cat#66562ST, CST), and vimentin (1:200, Cat#5741S, CST). For immune infiltration and fibrosis, anti-F4/80^+^ (1:50, Cat# 111101, Abcam), anti-CD4 (1:1000, Cat#11089; Abcam), and Col1a1 (1:200, Cat#72026S, CST) were used. Cellular senescence was determined by staining the prostate tissue slides with p21 (1:500, Cat#109199, Abcam) and p16 (1:500, Cat#51243, Abcam).

### 2.11. Western Blot

HPFs (50,000 cells/per well) were seeded in a 6-well plate. The next day, the cells were incubated with CM from obese and irradiated obese 3T3-L1 adipocytes for 96 h, with media replenishment at 48 h. After 96 h, the cells were lysed in lysis buffer containing 120 mM NaCl, 5 mM EDTA, 50 mM Tris-HCl, 1% NP-40 to prepare the whole cell extracts with complete protease inhibitor tablets (cat # 11697498001 Roche, Basel, Switzerland, 1 tablet/50 mL) as previously described [46,47]. The protein concentration was determined using a BCA kit and the quantified samples (20–30 µg per well) were resolved on a 4–12% BisTris gel (Cat # NW04120BOX, Thermo Fisher Scientific). The gel was then transferred to a nitrocellulose membrane (Cat # IB23002, Life Technologies, Carlsbad, CA, USA) and blocked with non-fat dry milk prepared in Tris-buffered saline with 0.1% Tween 20 (TBST). The blocked membranes were incubated at 4 °C with primary antibodies overnight. The next day, the membranes were washed three times with TBST and subsequently incubated with secondary antibodies (1:10,000 dilution) conjugated with horseradish peroxidase (HRP) for 1–2 h at room temperature. Blots were developed using chemiluminescence ECL (Thermo Fisher Scientific) and exposed to film. For loading control, Ponceau staining was done on total protein of each blot and the protein expression was normalized to its respective Ponceau staining. Protein expression was quantified using ImageJ. The following primary antibodies were used at the following concentrations: α-SMA (1:1000, Cat#NB300-978 Invitrogen), vimentin (1:1000, Cat#5741S, CST), FAP (1:1000, Cat#66562ST, CST), and Bcl-2 (1:1000, Cat#27476S, CST).

### 2.12. Quantitative RT-qPCR

HPFs (50,000 cells/per well) incubated in the presence or absence of the obese or irradiated obese 3T3-L1 adipocyte CM for 96 h, with media replacement at 48 h were collected for RNA isolation. Total RNA was isolated using an RNA isolation kit from Qiagen (Venlo, 74104, The Netherlands) as described previously [4]. The isolated RNA (1 µg) was converted into cDNA using an applied biosystems kit (Cat#4368814, Applied Biosciences, Waltham, MA, USA). RT-qPCR was performed using a SYBR Green Master Mix, and the following cycle conditions were used: 95 °C for 5 min, 30 s for 95 °C, 58 °C for 1 min, and 30 s at 72 °C for 39 cycles. The gene expression was normalized to RPLPO (housekeeping gene) (F-5′-GTCCTCGTGGAAGGCCC-3′, R-5′-AGGAGAGACAGGGAGCTCAG-3′). The data is presented as fold change as compared to respective control. The primers used in this study are: Col1a2 (F-5′-ACCATGGTGACCAGCGATAC-3′; R-5′-ACAAGGCATTCGTGGCGATA-3′), Col6a2 (F-5′-CTCCTCGGGACCAGGACTT-3′; R-5′-GGTGTCCAGCACGAAGTACA-3′), and Col3a1 (F-5′-TGTGTTTCGTGCAACCAT-3′; R-5′-CTTCTCTCCAGCCGAGCTTC-3′).

### 2.13. Transwell Migration Assay

All transwell migration experiments were performed using 8 μm tissue culture hanging inserts for tissue culture plate (Cat#PTE06H48, Millipore Sigma). 1.5 × 10^5^ MPFs, HPFs or prostate cancer cells (PC3, C42B) were seeded in 2% serum supplemented RPMI or DMEM media in the inserts for 48 h. In the lower chamber, there were normal MPFs or activated MPFs from obese or irradiated obese microenvironment in a 6-well plate. After 48 h, the migrated cells from the insert were trypsinized and the cells were scraped gently from the insert using a scraper and added to the media collected beneath the insert. The cells were centrifuged at 500× *g* for 5 min. The pellet was resuspended in 100 µL of fresh media and a small aliquot was mixed with trypan blue at a 1:1 ratio. The migrated cells were then counted using a hemocytometer.

### 2.14. Cytokine Array

Culture supernatants were collected from the MPFs treated with CM from obese or irradiated obese 3T3-L1 and from the normal MPFs (control), respectively. The collected CM from the respective conditions were centrifuged to eliminate debris at 500× *g* for 5 min and incubated with a pro-inflammatory cytokine array (Cat#ARY006, R&D Biosystems, Minneapolis, MN, USA) as per the manufacturers protocol.

### 2.15. ELISA

The conditioned media were collected from the normal prostate fibroblasts and fibroblasts exposed to obese or irradiated obese 3T3-L1 adipocyte CM. The CM was centrifuged to remove debris at 500× *g* for 5 min. The CM was diluted at a 1:1 ratio with the diluent provided in the ELISA kit of CXCL10 (Cat#ab260067, Abcam) and CXCL11 (Cat#ab204519, Abcam) and subsequent steps were performed as per the manufacturer’s protocol.

### 2.16. Bioinformatic Analysis

For bioinformatic analysis, the OncoDB platform (https://oncodb.org/) was used to perform correlation analyses of CXCL10, CXCL11, and CXCR3 gene expression in patients with prostate adenocarcinoma (PRAD). OncoDB is a publicly available cancer multiomics database that integrates data from The Cancer Genome Atlas (TCGA). The PRAD dataset included 505 tumor samples with gene expression data.

### 2.17. Statistical Analysis

For statistical analysis, GraphPad Prism v10 was utilized. The graphical data is represented as mean ± SEM unless otherwise indicated. For in vitro experiments, the experiments were repeated three to four times independently for statistical analysis. For changes in oxidative stress, senescence, inflammation, and fibrosis assuming a coefficient of variation of 0.4, alpha = 0.05 to detect a twofold change with 85% power, we estimated that we needed 5 mice. For in vivo experiments, at least five mice were included in each group and statistical analysis was done on the mean value obtained from the five mice per group. For statistical analysis, one-way ANOVA followed by Tukey’s post hoc multiple-comparisons test or unpaired Student’s *t*-test was used to determine the significance between multiple groups.

## 3. Results

### 3.1. Obesity Alone or in Combination with Radiation Induces Oxidative Stress and Inflammation

**Figure 1:**
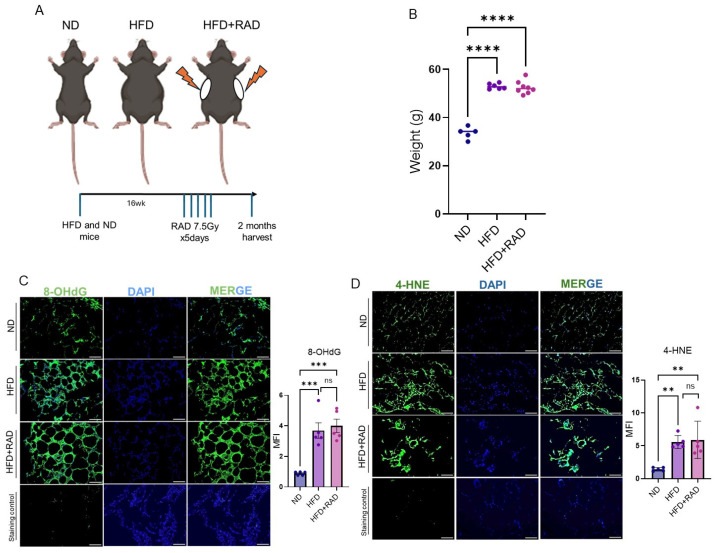
Obesity alone or in combination with radiation induces oxidative stress markers in adipose tissues. (**A**) Schematic representation of ND and HFD mice receiving direct irradiation of adipose tissue (7.5 Gy × 5 days) before harvest at 2 months post-radiation (ND, *n* = 5; HFD, *n* = 6; HFD+RAD, *n* = 8). (**B**) Weight of ND, HFD, and HFD mice at 2 months post-radiation. (**C**) Left, adipose tissue sections were stained with 8-OHdG (green color), an indicator of DNA/RNA oxidation. Right, quantification of 8-OHdG staining. (**D**) Left, representative staining of 4-HNE (green color), a marker of lipid peroxidation on adipose tissue from ND, HFD, and obese mice receiving direct radiation. Right, quantification of 4-HNE staining. A minimum of 5–6 images were captured and averaged for quantification per mouse, *n* = 5 mice/group. No primary antibody was used as a negative staining control. The data represents the mean ± SEM, statistical analysis was performed using one-way ANOVA followed by Tukey’s multiple-comparisons test. Statistical analysis between the HFD and the HFD+RAD groups was also performed using Student’s *t*-test. Images were taken at 200× magnification, and the white bar represents 100 µm. ** *p* < 0.01, *** *p* < 0.001, **** *p* < 0.0001.

Previously, we demonstrated that whole pelvic radiation exacerbates obesity-associated pathology, leading to the accumulation of chronic tissue damage [4]. For this study, we wanted to elucidate the role that obese and irradiated obese adipose microenvironments play in contributing to a pro-tumorigenic niche. To this end, C57BL/6 mice were maintained on either a normal diet (ND) or a high-fat diet (HFD) for 16 weeks, after which a small portion of gonadal adipose tissue was locally irradiated using a SARRP irradiator in some of the HFD mice. Mice received 7.5 Gy of X-rays for 5 consecutive days (total dose of 37.5 Gy, 3 Gy/min) (Figure 1A). Two months post-radiation, body weights were recorded to assess systemic metabolic alterations. Notably, no significant differences in body weight were observed between the adipose-irradiated HFD mice and the HFD mice (Figure 1B), indicating that adipose radiation did not alter overall body mass under these conditions. Next, we determined the effects that obesity and irradiation had on adipose tissue remodeling and markers associated with damage. Adipose tissue from normal or obese mice was stained with 8-hydroxy-2-deoxyguanosine (8-OHdG) to measure DNA/RNA oxidation and 4-hydroxynonenal (4-HNE) to study lipid peroxidation. Elevated staining of 8-OHdG and 4-HNE was observed in HFD mice alone as compared to ND mice and HFD mice following irradiation as compared to ND mice (Figure 1C,D). Interestingly, 8-OHdG and 4-HNE staining were similar between HFD mice alone and HFD mice 2 months after radiation (Figure 1C,D), suggesting that obesity-induced persistent oxidative stress is not further exacerbated by radiation. Constant accumulation of oxidative stress contributes to a pro-inflammatory and fibrotic microenvironment [48,49,50,51,52]. Increased weight gain is known to induce phenotypic alterations in adipocytes, characterized by immune cell infiltration and the formation of crown-like structures, as previously reported [4]. To evaluate macrophage and immune cell infiltration, adipose tissues from unirradiated ND or HFD and irradiated HFD mice were stained for total macrophages using the F4/80^+^ marker and T helper cells with the CD4^+^ marker. We observed a significant increase in F4/80^+^ macrophages in both HFD and irradiated HFD adipose tissues as compared to ND controls (Supplementary Figure S1A). CD4^+^ T cell infiltration was elevated in HFD adipose and irradiated HFD adipose tissue as compared to ND (Supplementary Figure S1B). Again, no significant differences were detected between HFD mice alone and irradiated HFD mice for either F4/80^+^ or CD4^+^ staining, suggesting that obesity-induced immune infiltration is sustained but not further enhanced with radiation (Supplementary Figure S1A,B). Activated macrophages secrete pro-inflammatory cytokines, chemokines, and growth factors that amplify immune cell recruitment and establish a chronic inflammatory milieu [53,54,55]. Collectively, these findings indicate that obese and irradiated obese adipose tissues exhibit sustained oxidative damage and chronic inflammation.

### 3.2. Obesity and Irradiation-Driven Adipose Remodeling Promote Benign Prostate Hyperplasia and Stromal Fibroblast Remodeling

**Figure 2:**
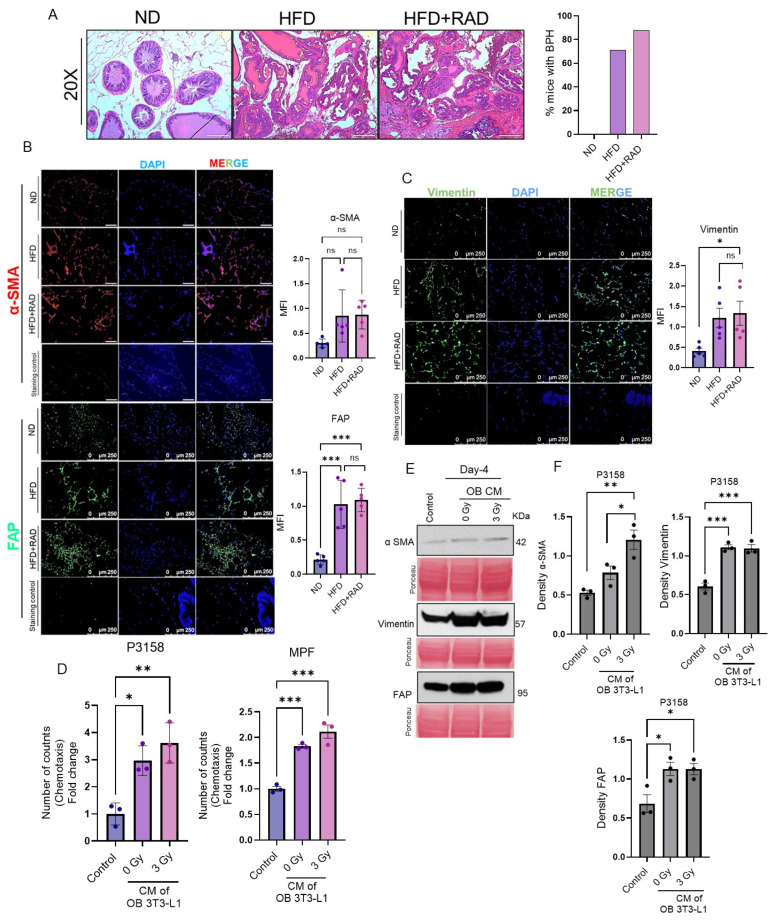
The obese or irradiated obese adipose microenvironment promotes benign prostate hypertrophy and stromal remodeling in vitro and in vivo. (**A**) Left, H&E images of prostate tissue from unirradiated and irradiated adipose of ND and HFD. Right, quantification of the percentage of mice with benign prostate hyperplasia (BPH). (**B**) Left, prostate tissue sections were stained with fibroblast activation markers including α-SMA (red color) and FAP (green color). Right, quantification of α-SMA and FAP. At least 5–6 images were taken per mouse and averaged for quantification (*n* = 5 mice/group). Data represents mean ± SEM. (**C**) Left, vimentin staining (green color), marker of mesenchymal cells on prostate tissue sections. Right, quantification of vimentin. (**D**) Normal mouse primary fibroblasts (MPFs) or human immortalized prostate fibroblasts P3158 cells were placed in the upper part of a Boyden chamber with conditioned media (CM) from obese and irradiated obese adipocytes placed in the bottom chamber to determine fibroblast motility. (**E**) Left, Western blot analysis of fibroblast activation markers in vitro on HPFs exposed to CM from unirradiated and irradiated obese 3T3-L1 CM for 96 hours (h). Ponceau was used as the loading control. Expression of α-SMA, FAP, and vimentin was normalized to their respective ponceau. Supplementary Figure S4 presents the original Western blot membranes. (**F**) Optical densitometry quantification of Western blot. Control conditions represent fibroblasts maintained in the standard RPMI or DMEM media. Data represents mean ± SEM from 3–4 biological independent experiments. Statistical analysis was determined using one-way ANOVA followed by Tukey’s multiple-comparisons test and Student’s *t*-test was also performed between the HFD and HFD+RAD groups. For immunohistochemical experiments, at least 5–6 images were captured per mouse and averaged for quantification, *n* = 5 mice/group. Images were captured at 200× magnification. The white bar represents 100 µm. * *p* < 0.05, ** *p* < 0.01, *** *p* < 0.001.

Myofibroblasts are an abundant part of the reactive stroma in the tumor microenvironment (TME), and influence cancer cell progression [34,56]. Cancer associated fibroblasts (CAFs) can directly interact with cancer cells through secreted cytokines and indirectly influence cancer progression by remodeling the ECM [37,57,58,59,60,61]. Given the established role of adipose-derived inflammatory and fibrotic mediators in stromal reprogramming [62], we wanted to determine the role of oxidatively stressed obese and irradiated obese adipose in promoting a pro-tumor microenvironment within the prostate. The H&E staining of prostate tissue from HFD and adipose-irradiated HFD mice demonstrated increased folding and multilayered epithelium along with enlarged acini, which are indicators of epithelial hyperplasia and early benign prostatic hyperplasia (BPH) as compared to prostates from ND mice (Figure 2A). BPH is accompanied by stromal remodeling [63]. Therefore, we evaluated myofibroblasts and fibroblast activation markers including α-smooth muscle actin (α-SMA, a myofibroblast marker), FAP (fibroblast activation marker), and vimentin (a myofibroblast marker) within the prostate tissue section from ND, HFD, and HFD mice that received radiation directly to the fat pads. We found elevated staining of α-SMA and FAP in the prostates from HFD mice alone, and irradiated HFD mice compared to ND mice; however, only FAP staining was significantly elevated (Figure 2B). Next, we also found increased vimentin staining in the prostate tissue section of irradiated HFD mice as compared to ND (Figure 2C). There was no significant difference in the fibroblast markers in prostate tissue of HFD and irradiated HFD mice (Figure 2B,C). The immunohistochemical analysis was performed on whole prostate tissue sections, and these markers cannot be attributed conclusively to prostate fibroblasts. Therefore, to investigate the role that obesity and radiation has on fibroblasts directly, we used normal MPF or immortalized human prostate fibroblast (HPFs). We found that CM from obese and irradiated obese adipocytes were able to chemoattract HPFs or MPFs within 48 h (Figure 2D). These changes are associated with fibroblast activation [64]. Subsequently, we also found that immortalized HPFs exposed to the CM from obese and irradiated obese adipocytes for 96 h expressed upregulated α-SMA, FAP, and vimentin (Figure 2E,F). Overall, these results indicate that an obese microenvironment can promote stromal remodeling within the prostate and promote a myofibroblast-like phenotype, likely via secreted factors from the obese adipocytes. However, irradiation of obese adipose tissue does not further amplify the stromal remodeling in prostate or prostate associated fibroblast markers in vitro but does maintain these phenotypes.

### 3.3. Obesity and Irradiation Promote Oxidative Stress and Survival of Prostate Fibroblasts In Vitro and In Vivo

**Figure 3:**
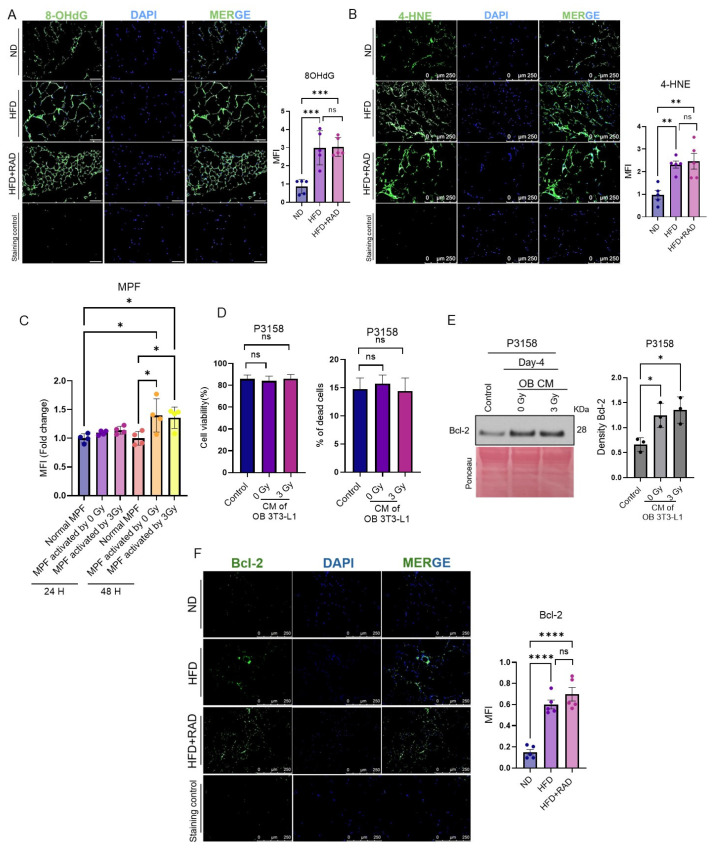
Prostate fibroblasts and prostate tissues are oxidatively stressed and express pro-survival factors in obese or irradiated obese adipose microenvironments in vitro and in vivo. (**A**,**B**) Left, prostate tissue sections from ND mice and HFD mice whose adipose was unirradiated or irradiated were stained for markers of oxidative stress including 8-OHdG (green color) and 4-HNE (green color). Right, quantification of the staining. (**C**) MPFs isolated from C57BL/6 mice were incubated with CM from irradiated or sham-control obese 3T3-L1 adipocytes for 96 h. The CM was replaced with fresh CM after 48 h. After 96 h exposure to CM from obese and irradiated obese 3T3-L1 adipocytes, MPFs were washed and incubated in fresh media for 24 h and 48 h for the estimation of H_2_O_2_ in vitro. A two-way ANOVA followed by a multiple-comparison test was used to determine statistical significance. (**D**) Immortalized human prostate fibroblasts were treated for 96 h with CM collected from sham control or irradiated obese 3T3-L1 adipocytes. CM was replenished after 48 h. At 96 h, cell viability and cell death were assessed by trypan blue exclusion assay. (**E**) Left, immunoblot of Bcl-2, a cell survival marker, in HPFs after 96 h of treatment with CM from obese and irradiated obese 3T3-L1 adipocytes, with media replaced after 48 h. Ponceau is used as loading control. Right, densitometry of immunoblot showing Bcl-2 expression normalized to the ponceau. Supplementary Figure S5 presents the original Western blot membrane. (**F**) Left, representative immunohistochemical images of Bcl-2 (green color) in prostate tissue sections of ND, and HFD mice alone and 2-months post-radiation of HFD mice. Right, quantification of the staining. For immunohistochemical experiments, at least 5–6 images were captured per mouse and averaged for quantification, *n* = 5 mice/group. HPF incubated in standard RPMI media served as the control condition. Images were captured at 200× magnification, and the white bar represents 100 µm. Data represents mean ± SEM from 3–4 biological independent experiments (in vitro) and statistical significance was determined by one-way ANOVA followed by Tukey’s multiple-comparisons test. Student’s *t*-test was also performed between the HFD and HFD+RAD groups to determine the statistical differences. * *p* < 0.05, ** *p* < 0.01, *** *p* < 0.001, **** *p* < 0.0001.

Obese or irradiated obsess adipose tissue enhances oxidative stress (Figure 1). Fibroblasts become activated either by secretory factors from the microenvironment or exposure to reactive oxygen species, which can promote a tumorigenic niche, including chronic tissue dysfunction, cellular senescence, inflammation, and fibrosis [42,64,65,66]. In our previous study, we reported that obese and irradiated obese adipocytes in vitro and in vivo are oxidatively stressed, release free fatty acids and hydrogen peroxide, and secrete pro-inflammatory adipokines including, C-X-C motif chemokine ligand 12 (CXCL12), interleukin-1 alpha (IL-1α), C-C chemokine ligand 2 (CCL2), tumor necrosis factor α (TNF-α), and transforming growth factor β (TGF-β) [4]. Therefore, we investigated whether the normal prostate tissue or prostate fibroblasts when exposed to obese or irradiated obese adipose tissue also become oxidatively stressed. We performed 8-OHdG and 4-HNE staining in the prostate tissue of unirradiated ND, HFD, and irradiated HFD mice. Elevated staining of 8-OHdG in HFD and adipose-irradiated HFD mice was observed in normal prostate tissue when compared to ND (Figure 3A). We observed no significant difference between the staining of 8-OHdG in HFD alone and irradiated HFD in the normal prostate tissue (Figure 3A). We also found enhanced 4-HNE staining in the HFD and irradiated HFD prostate as compared to ND (Figure 3B). Again, the staining of 4-HNE was similar between HFD and irradiated HFD mice (Figure 3B). These results suggest that prostates exposed to obese or irradiated obese adipose microenvironments are oxidatively stressed. However, as these analyses were performed on whole tissue sections, they do not specifically indicate whether prostate fibroblasts are oxidatively stressed. To address this, we performed in vitro experiments exposing normal MPFs to the CM from obese and irradiated obese adipocytes for 96 h, with media replenishment at 48 h. We determined the extracellular H_2_O_2_ production using an Amplex Red assay from the conditioned media of MPFs after 96 h exposure to the respective CM. We found a significant increase in the extracellular levels of H_2_O_2_ in the media of MPFs grown in an obese and adipose-irradiated obese microenvironment at 48 h but not at 24 h as compared to the CM from untreated MPFs (Figure 3C). Moderate or sustained extracellular H_2_O_2_ can promote stress adaptation and support cellular survival [67,68]. To examine this, we exposed normal immortalized human prostate fibroblasts to the CM from obese and irradiated obese adipocytes. After 96 h, cell viability was analyzed by trypan blue exclusion staining. Notably, the viability of HPFs treated either with obese or irradiated obese adipocyte CM was not altered (Figure 3D). Next, we examined the pro-survival protein Bcl-2 by Western blot. Consistent with the stress-adaptive phenotype, we observed HPFs exposed to the CM from obese and irradiated obese adipocytes for 96 h, showed higher expression of Bcl-2 as compared to normal HPFs (Figure 3E). The higher expression of Bcl-2 suggests pro-survival signaling that enables activated fibroblasts to withstand oxidative stress without undergoing cell death. Next, we validated these findings in vivo by staining the prostate tissue for Bcl-2. Immunohistochemical analysis revealed elevated Bcl-2 expression in both HFD and irradiated HFD groups compared to ND controls (Figure 3F). Overall, these results further support the notion that irradiated and obese adipose microenvironments enable a pro-survival phenotype in the prostate tissue and specifically in the prostate fibroblasts.

### 3.4. Prostate Fibroblasts Exhibit a Senescent-like Phenotype In Vitro and In Vivo When Exposed to Obese and Adipose Irradiated Obese Microenvironment

**Figure 4:**
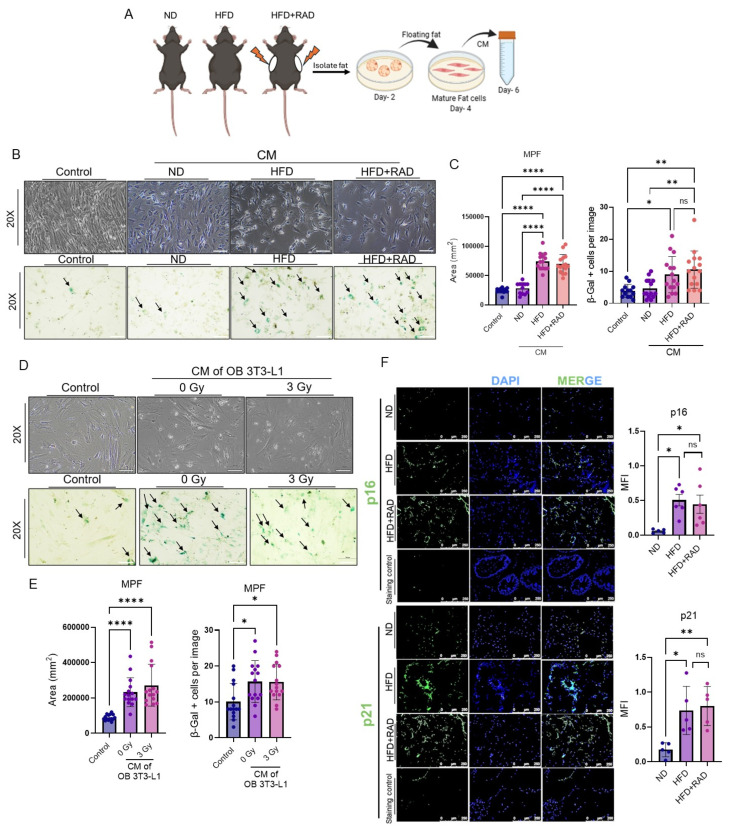
Prostate fibroblasts and prostate tissues become senescent in vitro and in vivo when exposed to an obese or irradiated obese adipose microenvironment. (**A**) Schematic of ex vivo collection of CM from adipose microenvironment isolated from unirradiated and irradiated obese mice. (**B**) Top, representative images showing the area of normal MPFs exposed to the ex vivo CM collected from the adipose of ND, HFD and irradiated HFD for 96 h to assess fibroblast activation. The ex vivo CM was replaced after 48 h. Bottom, representative images of MPFs showing the β-galactosidase staining after 96 h incubation with CM of ex vivo adipose from ND, HFD, and HFD+RAD groups. (**C**) Quantification of the MPF area and β-galactosidase-positive cells per image in each group. Area was calculated by tracing the borders of the images in ImageJ. 3–5 images were captured per biological replicate, *n* = 3. (**D**) Top, representative images of the area of normal MPF cultured with CM collected from unirradiated or irradiated obese 3T3-L1 adipocytes. CM was harvested 48 h after irradiation and applied to MPF for 96 h, with medium replacement at 48 h. Bottom, β-galactosidase staining of MPFs incubated with CM from obese and irradiated obese 3T3-L1 adipocytes for 96 h, with media replaced after 48 h. (**E**) Quantification of the area and number of β-galactosidase-positive cells of MPF per image in each group. 3–5 images were captured per biological replicate, *n* = 3. MPF cultured in DMEM media served as a control. (**F**) Left, representative images of cellular senescence markers (green color) in prostate tissue sections from unirradiated ND and HFD mice, as well as from irradiated HFD mice two months after radiation. Right, quantification of p16 and p21. At least 5–6 images were captured per mouse and averaged for quantification and statistical significance was calculated using one-way ANOVA followed by Tukey’s multiple-comparisons test. A statistical analysis between the HFD and HFD+RAD groups was also performed using Student’s *t*-test. Images were taken at 200× magnification, and the white bar represents 100 µm. Data represents the mean ± SEM. * *p* < 0.05, ** *p* < 0.01, **** *p* < 0.0001.

We have shown that prostate fibroblasts under obese and irradiated obese microenvironment maintain viability despite elevated oxidative stress. Therefore, we hypothesize that normal prostate fibroblasts might undergo stress adaptation in the form of cellular senescence rather than apoptosis or cell death. Previously, we have shown that whole pelvic radiation elevates senescence in obese and normal adipose tissues [4,27]. Senescence is characterized by the irreversible growth arrest of cells accompanied by altered secretory and metabolic state [69,70,71,72,73]. Therefore, we investigated markers of senescence in prostate fibroblasts when exposed to obese or irradiated obese microenvironment. We isolated mature fat cells directly from the adipose tissue of ND, HFD, and HFD mice that received direct radiation to the fat pads (Figure 4A). The CM was collected from the mature fat cells respectively (Figure 4A) and was then added to the normal prostate fibroblasts for 96 h, with media replenishment at 48 h (Figure 4B). We found MPFs exposed to the CM of HFD adipose or adipose-irradiated HFD adipocytes showed an increase in cell size along with increased β-gal staining as compared to either MPFs exposed to CM of normal adipocytes or standard culture media (control) (Figure 4B,C). Increased in the size of the fibroblasts demonstrates differentiation of fibroblasts to myofibroblasts and β-galactosidase staining indicates senescence [74]. Next, we validated these findings from the CM collected directly from obese and irradiated obese adipocytes. The MPFs incubated with the CM from obese and irradiated obese adipocytes also showed similar results as above with a significant increase in the MPFs cell size (Figure 4D,E) and β-gal staining as compared to the control (Figure 4D,E). Thus, suggesting prostate fibroblasts exposed to obese or irradiated obese adipocyte-derived CM acquire a senescence-like phenotype. Subsequently, we also validated these findings by performing immunohistochemical staining of senescent markers, including p16 and p21, on the whole prostate tissue sections of ND, HFD, and irradiated HFD adipose. Elevated staining of p16 and p21 was observed in the HFD and adipose-irradiated HFD prostates, as compared to control (ND) (Figure 4F). We did not observe any significant difference in the staining of p16 and p21 between the HFD and irradiated HFD prostates of obese and irradiated obese adipose microenvironments (Figure 4F). Since this analysis was performed on whole tissue sections, we cannot identify the specific cell type undergoing senescence. Collectively, these results indicate that obesity reprograms the prostate toward a senescent-like phenotype that can promote the maintenance of a tumor permissive microenvironment.

### 3.5. Prostate Fibroblasts Exposed to Obese or Irradiated Obese Adipose Microenvironments Acquire Pro-Inflammatory and Pro-Fibrotic Phenotypes In Vitro and In Vivo

**Figure 5:**
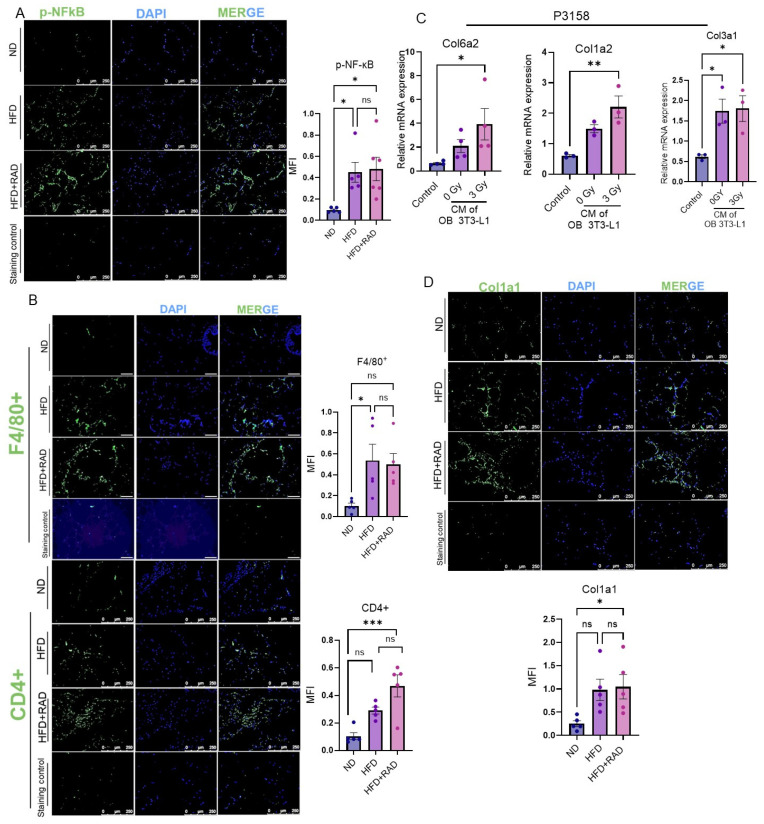
Prostate tissues from obese or irradiated obese adipose mice or prostate fibroblasts exposed to obese or irradiated obese CM exhibit pro-inflammatory and fibrotic phenotypes. (**A**) Representative images of p-NF-kB from the prostate tissue of unirradiated ND and unirradiated and irradiated adipose of HFD mice. Right, quantification of the p-NF-kB expression. (**B**) Representative images of prostate tissue from unirradiated ND and unirradiated and irradiated adipose of HFD mice stained with inflammation markers including F4/80^+^ (green color) and CD4^+^ (green color). Right, quantification of the F4/80^+^ and CD4^+^. For immunohistochemistry, 5–6 images were captured per mouse and were averaged for quantification. Statistical significance was calculated using a one-way ANOVA followed by Tukey’s multiple-comparisons test. Data represents the mean ± SEM (*n* = 5 mice/group) (**C**) RT-qPCR analysis of pro-fibrotic markers on HPFs treated with CM from obese and irradiated 3T3-L1 adipocytes for 96 h, with media replenishment after 48 h. Gene expression levels were normalized to housekeeping controls and expressed relative to control conditions. HPFs cultured in RPMI media served as the control condition. (**D**) Representative immunohistochemical images of Col1a1 staining (green color), a pro-fibrotic marker, in prostate tissue sections from ND and HFD mice alone and 2 months post-radiation, demonstrating increased fibrotic remodeling under obese and irradiated conditions. Bottom, quantification of Col1a1 staining. For immunohistochemical experiments, at least 5–6 images were captured per mouse and averaged for quantification, *n* = 5 mice/group. The white bar represents 100 µm. Images were captured at 200× magnification. For in vitro experiments, all the data were repeated 3–4 times independently. Data represents the mean ± SEM. Statistical significance was calculated using a one-way ANOVA followed by Tukey’s multiple-comparison test. Student’s *t*-test was also performed to compare the HFD and HFD+RAD groups. * *p* < 0.05, ** *p* < 0.01, *** *p* < 0.001.

Senescent stroma secretes inflammatory cytokines, chemokines, and extracellular remodeling factors that enforce chronic inflammation and fibrosis, which promote a pro-tumor microenvironment [75,76,77]. We then examined the whole prostate tissue section from ND, HFD, and irradiated HFD adipose microenvironment with NF-ĸB activation and immune cell infiltration markers, such as F4/80^+^ and CD4^+^. Activation of NF-ĸB is a central mediator of inflammatory signaling that recruits immune cells [53,78,79,80]. Increased staining of p-NF-ĸB was observed in the prostates of obese and adipose irradiated obese mice as compared to ND mice (Figure 5A). However, we did not observe a significant difference in the p-NF-ĸB staining between prostates from obese and adipose irradiated obese mice (Figure 5A). Elevated staining of F4/80^+^ was found in the prostate tissue of HFD as compared to ND prostate (Figure 5B). We observed a significant increase in the CD4^+^ staining in irradiated HFD prostate as compared to ND, but HFD did not induce a significant change as compared to ND (Figure 5B). Again, we found no significant difference in F4/80^+^ and CD4^+^ staining in the HFD and irradiated HFD prostates (Figure 5B). These results suggest that obesity with or without radiation creates pro-inflammatory stromal remodeling in the prostate. Chronic inflammation is a well-established driver of a fibrotic microenvironment [81,82,83]. Therefore, we measured fibrotic remodeling by immunohistochemical analysis and RT-qPCR. HPFs were exposed to either CM from obese or irradiated obese adipocytes for 96 h, with media replenishment at 48 h. A significant increase in the expression of Col6a2, Col1a2, and Col3a1 was observed in fibroblasts exposed to irradiated obese adipocyte CM as compared to normal HPFs (Figure 5C). However, no significant increase was found in Col6a2 or Col1a2 in HPFs exposed to obese adipocyte CM, although Col3a1 was significantly elevated (Figure 5C). Next, fibrotic remodeling within prostate tissue sections were evaluated by immunohistochemical staining for Col1a and α-SMA (Figure 2B). Elevated Col1a1 expression was observed in the whole prostate tissue sections of adipose-irradiated HFD mice as compared to ND (Figure 5D). We did not find any significant difference in the Col1a1 staining within prostates from HFD or irradiated HFD mice consistent with the above immunohistochemical results (Figure 5D). Although elevated inflammatory and fibrotic markers were observed in the whole prostate tissue sections, these analyses do not allow the definitive identification of the specific cell type responsible for these changes. However, our in vitro data strongly supports that prostate fibroblasts which have transitioned into myofibroblasts, acquire a pro-fibrotic phenotype under obese and irradiated obese adipocyte conditions. Overall, these results suggest that the irradiated obese microenvironment enhances inflammation and fibrosis as compared to normal conditions.

### 3.6. Prostate Fibroblasts Exposed to Obese or Irradiated Obese Adipose Microenvironment Promote Prostate Cancer Progression

**Figure 6:**
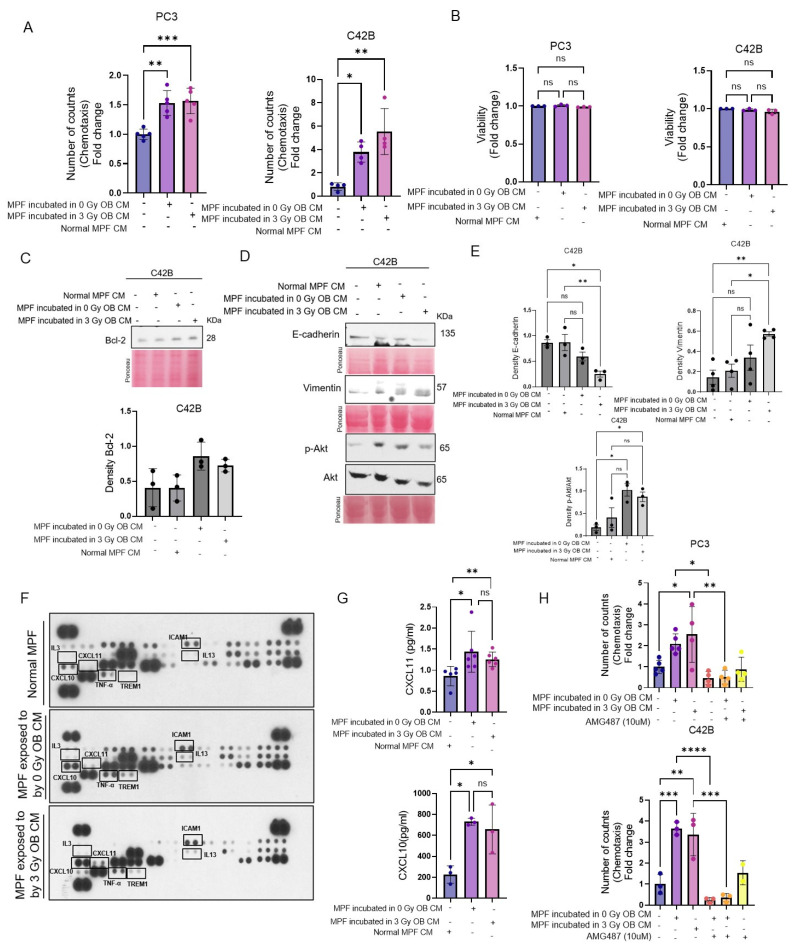
Prostate fibroblasts exposed to an obese or irradiated obese adipose microenvironment promote prostate cancer progression. (**A**) Migration assay of PC3 and C42B toward the MPF exposed to obese and irradiated obese 3T3-L1 adipocyte CM and normal MPF CM for 48 h. (**B**) Cell viability of PC3 and C42B when exposed to MPF CM incubated in obese and irradiated obese 3T3-L1 adipocytes for 48 h. (**C**) Top, Western blot analysis of the cell survival marker, Bcl-2, on C42B cells incubated with normal MPF CM and MPF CM exposed to obese and irradiated obese 3T3-L1 adipocytes for 48 h. Ponceau staining was used as the loading control. Bottom, densitometry of immunoblot showing Bcl-2 expression normalized to the corresponding Ponceau staining. Supplementary Figure S6 represents the original Western blots. (**D**) Western blot analysis of EMT markers and p-Akt in C42B cells after 48 h of exposure to CM from MPFs incubated in obese and irradiated obese 3T3-L1 adipocytes and from normal MPFs. Ponceau was utilized as loading control. Supplementary Figure S7 represents the original membranes of Western blot. (**E**) Densitometry of EMT markers and p-Akt in C42B normalized to ponceau. (**F**) Cytokine array of culture supernatants collected from the MPF exposed to obese and irradiated obese 3T3-L1 adipocytes for 96 h, with media replenishment at 48 h. After 96 h, the CM from obese and irradiated obese adipocytes was removed from MPFs from the respective conditions and washed with PBS 3 times. After 24 h of culture, the fresh MPF medium was collected from the respective treated conditions and normal conditions *n* = 1. The original membrane of cytokine array is represented in Supplementary Figure S8. (**G**) CXCL11 and CXCL10 ELISAs performed on the CM collected from normal MPF and fibroblasts exposed to obese and irradiated obese 3T3-L1 adipocytes. (**H**) Transwell migration assay of PC3 and C42B demonstrating the functional role of CXCR3 signaling in PCa migration toward the MPF exposed to obese and irradiated obese 3T3-L1 adipocytes. All in vitro data are presented as mean ± SEM from four or more biological independent experiments except (**F**). PCa cells incubated in standard RPMI culture served as a control. Statistical significance was determined by one-way ANOVA followed by a Tukey’s multiple-comparison test. * *p* < 0.05, ** *p* < 0.01, *** *p* < 0.001, **** *p* < 0.0001.

**Figure 7:**
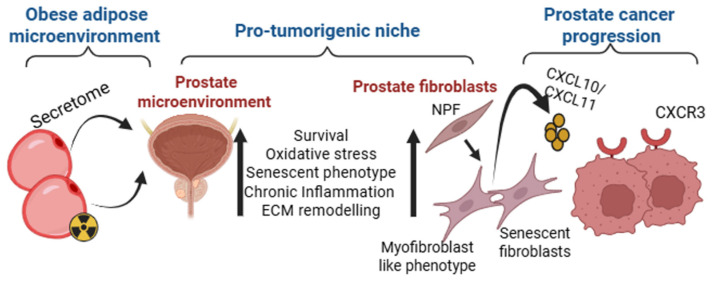
Overview of obesity and radiation associated adipose dysfunction promotes a pro-tumorigenic prostate microenvironment. This stress-adapted phenotype is characterized by enhanced survival signaling, oxidative stress, chronic inflammation, senescence-associated remodeling, and ECM remodeling, collectively establishing a tumor-permissive stromal niche. The obese and irradiated obese adipose microenvironment differentiates prostate fibroblasts toward myofibroblasts and senescent fibroblasts. In turn, myofibroblasts or senescent fibroblasts under obese and irradiated obese adipose microenvironments secrete pro-inflammatory chemokines, including CXCL10 and CXCL11, which promote prostate cancer migration through CXCR3-mediated paracrine signaling. Radiation exposure sustains obesity-associated pathological state within the prostate microenvironment and prostate fibroblasts.

Myofibroblasts are known to promote different cancers including pancreatic, lung, colon, and prostate cancer through the secretion of growth factors and inflammatory mediators [41,84,85,86]. Given our findings that normal prostate fibroblasts transition to myofibroblasts under obese and irradiated obese conditions, we next investigated whether these prostate myofibroblasts promote prostate cancer progression. Therefore, we performed a transwell migration assay and found that prostate cancer cells (PC3 and C42B) exhibited enhanced migration toward the CM of prostate fibroblasts incubated with obese and irradiated obese adipocytes, as compared to the normal prostate fibroblasts (Figure 6A). However, no significant differences in the migratory responses were observed between fibroblasts incubated with obese versus irradiated obese conditions (Figure 6A). Although prostate fibroblasts under obese and irradiated obese conditions did not alter the viability of PCa cells (Figure 6B), we observed a non-significant trend of elevated pro-survival protein expression of Bcl-2 in PCa cells exposed to the CM from prostate fibroblasts that transitioned into myofibroblasts as compared to normal prostate fibroblasts and cells incubated in standard culture media (control), suggesting a potential survival advantage (Figure 6C). Because prostate fibroblasts exposed to obese and irradiated obese adipocytes enabled PCa migration, we next examined whether they induce epithelial-to-mesenchymal transition (EMT) changes in PCa cells. Incubation of C42B cells with CM from prostate fibroblasts that had been exposed to CM from obese adipocytes, irradiated obese adipocytes, and normal prostate fibroblasts for 48 h resulted in decreased in the expression of E-cadherin, an epithelial cell marker, as compared to control and normal prostate fibroblasts (Figure 6D,E). We also observed a significant upregulation in the mesenchymal marker, vimentin, under irradiated obese activated MPF CM as compared to PCa cells incubated in standard culture media (Figure 6D,E). Similarly, a decrease in E-cadherin and an increase in vimentin was observed in PC3 cells incubated with CM from MPFs that transitioned into myofibroblasts when exposed to obese and irradiated obese adipocytes as compared to standard culture media or normal prostate fibroblasts (Supplementary Figure S2). Moreover, CM from prostate fibroblasts exposed to obese and irradiated obese adipocytes significantly increased the phosphorylation of Akt (p-Akt), which is implicated in cell migration and tumor progression, compared with control conditions (Figure 6D,E). Collectively, these findings suggest that prostate fibroblasts under obese and irradiated obese conditions promote EMT-associated reprogramming and Akt elevation in PCa cells. To identify factors contributing to enhanced PCa migration, we performed a pro-inflammatory cytokine array using CM collected from normal and prostate fibroblasts exposed to an obese and irradiated obese adipocyte. Prostate fibroblasts incubated with obese and irradiated obese conditions displayed increased secretion of pro-inflammatory cytokines including CXCL11, CXCL10, IL-13, TREM1, and TNF-α, all of which are known to induce different cancers including hepatocellular carcinoma, colon cancer, and gastric cancer [37,40,87,88,89]. Because CXCL10 and CXCL11 were among the most highly upregulated factors secreted by prostate fibroblasts exposed to obese and irradiated obese adipocyte conditions, we validated these findings by performing ELISAs. Notably, we found higher secretion of CXCL10 and CXCL11 from fibroblasts exposed to obese and irradiated obese adipocyte CM as compared to normal prostate fibroblasts (Figure 6G). Therefore, we focused on their potential role in mediating fibroblast-driven PCa migration. As a member of the CXC chemokine family, CXCL10 and CXCL11 exert their function by binding to the CXCR3 receptor, and we found both CXCL11 and CXCL10 displayed a positive correlation with CXCR3 (Supplementary Figure S3). This positive interaction has been implicated in cellular processes such as tumor progression and cell migration of various cancers including prostate, colon, and head and neck cancer [90,91,92,93]. To validate the functional role of CXCL10/CXCL11-CXCR3 paracrine signaling axis, PCa cells were pre-treated with the CXCR3 inhibitor, AMG-487, prior to migration assays. AMG487 has been extensively characterized and widely used as a selective CXCR3 inhibitor in previous studies [40,94]. Pharmacologic inhibition of CXCR3 markedly reduced the migration of both PC3 and C42B cells toward CM from fibroblasts exposed to obese and irradiated obese conditions (Figure 6H), supporting a paracrine role for this signaling axis in promoting PCa cell motility. Together, these findings demonstrate that prostate fibroblasts that exhibit a myofibroblast-like phenotype under obese and irradiated obese adipose microenvironments enhance PCa migratory potential, promote EMT-associated signaling, and may drive tumor-promoting paracrine communication through the CXCL10/CXCL11-CXCR3 axis (Figure 7). These results identify that this pathway may serve as a therapeutic target against CXCL10/CXCL11-driven prostate cancer tumor progression.

## 4. Discussion

Obesity is recognized not only as a metabolic disorder but, importantly, there is growing evidence that obesity is associated with cancer incidence, recurrence, and enhanced mortality [95]. A major component of the tumor microenvironment includes stromal fibroblasts, which are principal contributors to cancer progression [96]. When stromal fibroblasts acquire a myofibroblast phenotype, they secrete chemokines and growth factors contributing to the metastatic potential of various cancers [34,56,97]. Radiation therapy is the first line of treatment for various cancers. However, normal tissues surrounding pelvic cancer receiving radiotherapy are not spared from the detrimental effects of radiation. We have previously shown that abdominal irradiation promotes the damage to the adipose tissue present within the pelvic region of obese mice [4]. Specifically, abdominal irradiation in the HFD mice reduced the body weight and increased oxidative stress, senescence, inflammation, and fibrosis in the adipose tissue. In contrast, in the present study, the direct and partial radiation to the gonadal fat pad did not alter the body weight of irradiated HFD mice and did not elevate the oxidative stress and inflammation within adipose tissue; rather, it maintained the obesity-induced adipose dysfunction.

This is the first study to investigate the role of radiation and obesity in promoting adipose dysfunction that reshapes the prostate microenvironment toward a pro-tumorigenic environment, at least in part through myofibroblasts. Our findings demonstrate that obesity and radiation influence the neighboring prostate tissue through chronic tissue remodeling characterized by the establishment of senescence, oxidative stress, inflammation, and fibrosis. We additionally observed that obesity and radiation increased epithelial hyperplasia and stromal associated markers.

Notably, the obese and irradiated obese adipocytes in vitro induced robust changes in the prostate fibroblasts, including elevation in α-SMA, FAP, vimentin, and senescence. Radiation exposure in the obese adipocytes did not further elevate myofibroblast markers in prostate fibroblasts and senescence. This is probably due to the fact that once fibroblasts differentiate into myofibroblast or senescent fibroblast by obesity, radiation cannot further differentiate these cells. However, in vivo, the irradiated obese adipose microenvironment did elevate the ECM remodeling, and infiltration of CD4^+^ T-helper cells as compared to normal conditions that were not altered by obesity. This indicates that radiation may further contribute to the pro-tumor microenvironment in vivo.

A key translational finding in this study is that MPFs exposed to obese or irradiated obese adipose conditions promote PCa cell migration, EMT, and AKT signaling. These findings align with previous studies demonstrating that myofibroblasts support tumor progression via paracrine communication and extracellular remodeling [37,41,98,99]. Because senescent fibroblasts exhibited similar phenotypes under obese and irradiated obese conditions, their effects on cancer progression were also similar. Mechanistically, we identified the CXCL10/CXCL11-CXCR3 signaling axis as a major pathway mediating myofibroblast-induced PCa migration, highlighting a direct link between adipose dysfunction, fibroblast phenotypic changes, and tumor progression.

Some limitations of this study are that, although our in vivo analyses demonstrate stromal remodeling within the prostate tissue, several markers assessed by IHC cannot exclusively be attributed to fibroblasts. Even though our in vitro studies address this limitation by demonstrating fibroblast specific responses to adipocyte-derived factors we did not show this conclusively in vivo. Secondly, we have addressed that irradiated obese adipocytes activate MPFs, but what adipocyte-derived factors or specific ROS that cause this phenotype has not been elucidated and requires mechanistic investigation. Third, we only focused on the effect of white adipose tissue on the prostate microenvironment, other fat tissues may respond differently to obesity and radiation and might show different pathological effects on the prostate microenvironment. Fourth, while our data implicates oxidative stress, senescence, inflammation, and fibrosis as interconnected processes within prostate microenvironment, it is important to determine the relationship of these pathways. Fifth, how CXCL10/CXCL11 is elevated during obesity or radiation in obese conditions requires further investigation. Sixth, future studies are required to incorporate localized irradiation to the gonadal fat pad of ND mice to determine the independent contribution of radiation alone on adipose tissue. Seventh, in the present study, only a small portion of obese adipose tissue was irradiated using the 5 × 5 mm collimator. Since the adipose tissue in HFD mice is triple the size of that in ND mice, the irradiated field in the HFD mice represented a small proportion of the total adipose depot. In our previous studies, HFD mice that received pelvic radiation showed more robust phenotypic changes as compared to unirradiated HFD mice, suggesting that irradiating a larger adipose area may produce more pronounced phenotypic effects. Therefore, future studies are required to irradiate a larger area of adipose tissue in HFD mice to accurately determine the role of radiation in promoting a pro-tumor microenvironment and disease progression in obesity. We also acknowledge that the CM from normal adipocytes would strengthen the experimental framework and align the in vitro observations with the in vivo comparison between ND and HFD. Lastly, our irradiation model to adipose tissue does not recapitulate the clinical setting of prostate radiotherapy where both the surrounding tissue and prostate receive radiation exposure.

In conclusion, our findings demonstrate that obesity-associated adipose dysfunction induces persistent oxidative stress, inflammatory signaling that drives changes in prostate fibroblast including myofibroblast differentiation, survival adaptation, senescence, and fibrotic signaling in vitro and pro-tumorigenic remodeling within the prostate. Prostate fibroblasts exhibiting a myofibroblast-like or senescent phenotype establish a chronic inflammatory and fibrotic microenvironment that promotes prostate cancer progression through paracrine signaling, particularly via the CXCL10/CXCL11-CXCR3 axis. An interesting finding from our study is that radiation exposure of obese adipose tissues does not significantly enhance these effects within the prostate microenvironment and on prostate fibroblasts but rather sustains the pre-existing pathological state. These results help us to explain the previously established evidence of association of obesity with poor outcomes and recurrence following radiotherapy [100,101]. Overall, these findings identify obese adipose-stromal crosstalk as an important regulator of prostate tumor-promoting microenvironments and suggest targeting fibroblast mediated paracrine signaling, particularly the CXCL10/CXCL11-CXCR3 axis. Further, in vivo studies are needed to determine whether targeting this pathway limits PCa tumor progression and improves therapeutic responses.

## 5. Conclusions

Obesity-associated adipose dysfunction drives pathological changes within the prostate microenvironment and differentiates prostate fibroblasts to myofibroblasts that are characterized by elevated survival signaling, oxidative stress, senescence, inflammation, and fibrotic remodeling. Radiation exposure to the obese adipose microenvironment does not further enhance the pro-tumorigenic microenvironment and differentiation of prostate fibroblasts to myofibroblasts rather, it sustains the obesity-associated pathological state. Additionally, prostate fibroblasts exhibiting myofibroblast-like or senescent phenotype under obese and irradiated obese adipose microenvironment promote prostate cancer progression through the CXCL10/CXCL11-CXCR3 signaling axis. Overall, our findings highlight obese adipose-stromal crosstalk as an important regulator of the prostate tumor microenvironment and could be a potential target to treat prostate cancer.

## Data Availability

The original contributions presented in this study are included in the article/Supplementary Material. Further inquiries can be directed to the corresponding author.

## Supplementary Materials

The following supporting information can be downloaded at: https://www.mdpi.com/article/10.3390/cells15151387/s1. Figure S1: Obesity alone or combined with radiation induces pro-inflammatory stress markers; Figure S2: Conditioned media from fibroblasts incubated with obese or irradiated obese adipocytes promoted EMT in PC3; Figure S3: Correlation analysis of CXCR3 with CXCL10/CXCL11; Figure S4: Original blots and loading control of Figure 2E; Figure S5: Original blots and loading control of Figure 3E; Figure S6: Original blots and loading control of Figure 6C; Figure S7: Original blots and loading control of Figure 6D; Figure S8: Original blot of Figure 6F.

## Abbreviations

The following abbreviations are used in the manuscript.

TME	tumor microenvironment
MPF	mouse prostate fibroblast
HPF	human prostate fibroblast
NPF	normal prostate fibroblast
HFD	high-fat diet
CM	conditioned media
PCa	prostate cancer
NEAA	non-essential amino acids
P/S	penicillin/streptomycin
ND	normal diet
BPH	benign prostate hyperplasia
α-SMA	alpha smooth muscle actin
ECM	extracellular matrix
FAP	fibroblast activation protein
8-OHdG	8-hydroxy-2-deoxyguanosine
4-HNE	4-hydroxynonenal
EMT	epithelial-to-mesenchymal transition
DMEM	Dulbecco minimal essential media
FBS	fetal bovine serum
CST	cell signaling technology
IACUC	Institutional Animal Care and Use Committee
SA-β-Gal	senescence-associated beta galactosidase
p-Akt	phosphorylation of Akt
SARRP	small animal radiation research platform
CXCL12	C-X-C motif chemokine ligand 12
IL-1α	interleukin-1 alpha
CCL2	C-C chemokine ligand 2
TNF-α	tumor necrosis factor α (TNF-α)
TGF-β	transforming growth factor β
